# Resolution-Enhanced Harmonic and Interharmonic Measurement for Power Quality Analysis in Cyber-Physical Energy System

**DOI:** 10.3390/s16070946

**Published:** 2016-06-27

**Authors:** Yanchi Liu, Xue Wang, Youda Liu, Sujin Cui

**Affiliations:** State Key Laboratory of Precision Measurement Technology and Instruments, Department of Precision Instrument, Tsinghua University, Beijing 100084, China; liuyanchi13@mails.tsinghua.edu.cn (Y.L.); liu-yd11@mails.tsinghua.edu.cn (Y.L.); cuisj14@mails.tsinghua.edu.cn (S.C.)

**Keywords:** harmonics, interharmonics, resolution-enhanced, independent component analysis, adaptive linear neuron, power quality, cyber-physical energy system

## Abstract

Power quality analysis issues, especially the measurement of harmonic and interharmonic in cyber-physical energy systems, are addressed in this paper. As new situations are introduced to the power system, the impact of electric vehicles, distributed generation and renewable energy has introduced extra demands to distributed sensors, waveform-level information and power quality data analytics. Harmonics and interharmonics, as the most significant disturbances, require carefully designed detection methods for an accurate measurement of electric loads whose information is crucial to subsequent analyzing and control. This paper gives a detailed description of the power quality analysis framework in networked environment and presents a fast and resolution-enhanced method for harmonic and interharmonic measurement. The proposed method first extracts harmonic and interharmonic components efficiently using the single-channel version of Robust Independent Component Analysis (RobustICA), then estimates the high-resolution frequency from three discrete Fourier transform (DFT) samples with little additional computation, and finally computes the amplitudes and phases with the adaptive linear neuron network. The experiments show that the proposed method is time-efficient and leads to a better accuracy of the simulated and experimental signals in the presence of noise and fundamental frequency deviation, thus providing a deeper insight into the (inter)harmonic sources or even the whole system.

## 1. Introduction

Cyber-physical energy system (CPES) is about the intersection of the physical and the cyber in the power grid [1,2]. Specifically speaking, not only the utilities can monitor the consumers’ power information and make corresponding operations such as providing suggestions for consumption reducing [3] and disturbances filtering [4], but also the consumers have the choice of the power consuming behaviors or even proposing demands to utilities, according to the utility-published data such as the dynamic price and power quality.

The main supporting technologies of achieving the above functionalities are two-way communication with multi-mode data transmission, dynamic demand response, and power quality analysis in the appliance level [3,5,6]. In this sense, the fundamental hardware that best connects the physical with the cyber is called a smart meter [6]. However, existing smart meters are not truly smart, only parameters such as active/reactive power, power consumption, root mean square of voltage and current are acquired, and these electric data are gathered every few minutes or hours [6]. Therefore, smart meters that can provide waveform-level information for power quality analysis are needed, and in terms of the communication, the power quality analysis framework should be carefully designed.

In CPES, power quality analysis is not restricted to the classic problems such as the definition and classification of disturbances, and measurement protocols and standards [7,8] have also been well established. Therefore, building tools for extracting the fine-grained information inside the power quality data accurately is of great interest to power quality researchers. In this paper, we take the harmonic distortion, which is the most common type of power quality disturbance, for example, low-order harmonics have been extensively discussed in the literature [9,10,11] because they contribute the most to related problems such as overheating the rotating machines and degrading the performance of electronic equipment in the traditional power system. However, as more renewable energy sources are put into use and microgrids are becoming widespread across the country, excessive interharmonic pollution has been injected into the system [12], which may indicate many characteristics of the power equipment within the system [13], and problems caused by frequency deviation and noise are also getting severe [4]. Therefore, compared to the methods only aiming at low-order harmonics and regardless of the frequency and noise, resolution-enhanced harmonic and interharmonic measurement can provide a more comprehensive understanding of the power quality.

Frequency estimation is an important factor to harmonic and interharmonic measurement [14,15]. The standard IEC (International Electrotechnical Commission) 61000-4-7 [8] recommends Fast Fourier Transform (FFT) and the subgroup method as the tools measuring the harmonics and interharmonics, with 5 Hz as the frequency resolution. However, the frequency of interharmonics is not an integer of the fundamental frequency, and fundamental frequency deviation is unavoidable in general; therefore, harmonic and interharmonic measurement needs higher frequency resolution. Additionally, noise and time-varying nature of the power signals should also be considered in the design of the measuring devices and algorithms. Distributed and advanced sensors equipped with advanced algorithms bring us a deeper and more accurate comprehension of the whole system [15,16,17,18,19,20]; however, they also make us confront a tradeoff between accuracy and computational complexity. High-resolution methods usually need considerable computational cost, thus mostly applied in off-line processing, and low-resolution methods such as FFT-based methods are more likely to be implemented in real-time hardware in contrast. In recent years, much attention has been devoted to harmonic and interharmonic estimation, and many methods have been proposed to achieve a better tradeoff [21,22,23].

Conventional methods for harmonic and interharmonic analysis can be divided into two categories: parametric and non-parametric methods. In contrast to non-parametric methods, parametric methods such as multiple signal classification (MUSIC), the Prony method, estimation of signal parameters via rotational invariance techniques (ESPRIT) [24,25,26,27] achieve higher frequency resolution and is more suited for the measurement of interharmonic. However, accurate estimation of these algorithms highly depends on the prior information of the model order, and the algorithms are time-consuming and not robust to noise and outliers in general. Discrete Fourier transform (DFT) as the most commonly applied non-parametric method is time-efficient and robust enough; however, its frequency resolution is restricted by the observation interval, and its result may be deteriorated by spectral leakage caused by interharmonic and fundamental frequency deviation [28].

Modern methods based on artificial neural networks (ANN) [9,10], adaptive linear neuron (ADALINE) network [11,21], independent component analysis (ICA) [29,30] and empirical mode decomposition (EMD) [31,32] have emerged in this field recently. ANN and ADALINE can be applied in real-time for time-varying power signals; however, traditional ADALINE and ANN methods are only capable of calculating the harmonic component’s amplitude and phase angle with the frequency known a priori, and noise and interharmonics have to be pre-filtered for accuracy, thus limiting the measurement of interharmonics. ICA-based methods formulate the harmonic and interharmonic estimation into a single-channel independent components extraction problem, and extract pure sinusoidal signals for subsequent processing, in order to reduce the computation burden. [30] proposes to leave the computation to the design stage to obtain the best separation row offline, and achieves accurate estimation results at the sacrifice of the adaptability to noise. EMD-based methods such as improved EMD with masking signals (IM-EMD) [32] also aim at extracting single-frequency harmonics from distorted time-varying power signals, but the masking parameters for IM-EMD are not consistent in different conditions and lack of self-adaption.

Considering the tradeoff between accuracy and computational complexity, numerous improvements for the above methods have been offered. One method alone has its intrinsic weakness, and thus hybrid methods like two-stage ADALINE [21], exact model order ESPRIT (EMO-ESPRIT) [33] and ESPRIT-assisted adaptive wavelet neural network (EA-AWNN) [22] have been proposed to compensate for each other’s weakness, thus achieving accuracy and time-efficiency simultaneously. Two-stage ADALINE integrates Prony method to help ADALINE locate every frequency component accurately. EMO-ESPRIT utilizes a model order estimation algorithm to provide prior information to ESPRIT for accurate estimation. To handle time-varying signals with higher accuracy, EA-AWNN leverages ESPRIT’s accurate estimation results to train the adaptive wavelet neural network in real-time. Furthermore, principles of compressive sensing have also been applied to DFT-based waveform analysis (CS-DFT) for higher frequency resolution [23], though the estimation accuracy is restricted by discrete-valued frequency estimates. The main idea of these methods is to first estimate the frequency using high-resolution methods, and then to estimate the amplitude and phase angle with fast and adaptive techniques.

This paper gives a detailed description of the power quality analysis framework in networked environment and presents a fast and resolution-enhanced method for harmonic and interharmonic measurement. It is based on the single-channel version of Robust ICA [34,35] (SC-RICA) to extract harmonic and interharmonic components efficiently at first, then leveraging the results computed by the previous stage. The high-resolution frequency is estimated from three DFT samples [36,37] with little additional computation, and, finally, the amplitudes and phases are calculated using the ADALINE [21] network. The experiments show that the proposed method is time-efficient and leads to a better accuracy of the simulated and experimental signals, and thus provides a deeper insight of the (inter)harmonic sources and even the whole system. The superiority of the proposed method is compared with those researched before, and the interference caused by fundamental frequency deviation and the presence of noise are also considered.

The remainder of this paper is structured as follows. Section 2 introduces the power quality analysis framework in networked environment. Section 3 formulates the problem and presents the basic principle of single-channel ICA (SCICA) and ADALINE. Section 4 describes every stage of the proposed method, and how the single-channel version of RobustICA assists ADALINE in extracting harmonic and interharmonic components from distorted power signals. The simulated and laboratory experiments are presented in Section 5 to evaluate the performance of the proposed method, the distorted power signals are acquired using a prototype system, and high-resolution methods such as EMO-ESPRIT, MUSIC and CS-DFT are used for comparison. Finally, the conclusion is given in Section 6.

## 2. Analysis Framework

As discussed in Section 1, existing smart meters are mainly used for billing purposes and demand monitoring, and due to the lack of computing resources and waveform measurement devices, they still cannot fulfill the demands such as power quality analysis in appliance level and the interaction between computerized instrumentation and physical facilities. Therefore, metering devices should be modified to make them truly smart and be dispersed throughout the system. Inspired by the work of nonintrusive load monitoring (NILM) initiated by George W. Hart [38], a nonintrusive, high-performance monitor can be connected with the total load using the standard revenue meter socket interface. This extension of the meter leads to very easy installation, removal, and maintenance, and there is no need to build lines especially for power quality analysis.

Wireless sensor networks (WSNs) are the most promising technology that connects the “last mile”, which refers to the connection that provides substations and consumers the access to high-speed and wide-bandwidth core network [39]. Wireless communication technologies help achieve remote control and monitoring without cable cost and wired communication infrastructures. Among the above technologies, cellular networks (3G/4G) and World interoperability for Microwave Access (WiMAX) are widely adopted in Neighborhood Area Networks (NANs), IEEE (Institute of Electrical and Electronics Engineers) 802.11 (Wi-Fi) is broadly used in Local Area Networks (LANs), the growing use of IEEE 802.15.1(Bluetooth) helps connect the Personal Area Networks (PANs), and IEEE 802.15.4 (ZigBee) may become the best choice for WSNs due to its low power consumption, low cost and self-organization [17,18,40,41]. Leveraging the advantages of WSNs, distributed sensing and state estimation can be easily realized, and sensors will cooperate against the inherent communication noise or packet losses to achieve a more robust system-wide observation [2,42,43].

**Figure 1:**
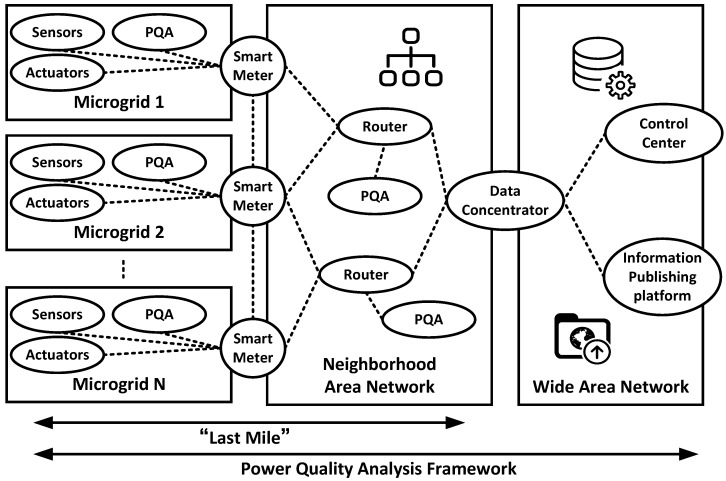
Power quality analysis framework in networked environment.

According to the above analysis, the design of the framework for power quality analysis in networked environment is depicted in Figure 1. Power quality analyzers (PQAs), as the extensions of the existing smart meters, should be connected in parallel with the smart meters using the same socket interface. Voltage and current waveforms are acquired and the power-quality-related computing is done by PQAs. Unlike traditional power systems, in this framework, PQAs are not only deployed at the point of common coupling, such as substations, but also at the lower level such as residential users, commercial users, and industrial users, and all the users can be regarded as micro-grids in the future energy system [4]. As for the smart meters, they mainly act as the agency between users and suppliers. The communication protocol for smart meters should be well-designed, for example, safety-critical information such as severe harmonic distortions or lightning impulses should be transmitted at a high reporting rate and should be high priority. The billing data and demand responses, meanwhile, can be delayed to some extent. After data acquisition, the data are transmitted through the wireless network in Neighborhood Area Networks and aggregated to the data concentrator, then the data are further processed in the control center. Furthermore, the control center can also be considered to be an information publishing platform, which is capable of announcing power-quality-related alerts or advice.

## 3. Theoretical Background

It is assumed that the power signal can be transformed to the frequency domain with the line spectrum, and the frequency components are finite and sparse. In this section, the high-frequency-resolution harmonic and interharmonic estimation will be disassembled into three stages: harmonic and interharmonic extraction, frequency estimation, amplitude and phase angle estimation. The harmonic and interharmonic extraction stage will be formulated into a single-channel blind source separation problem, and the amplitude and phase angle estimation will be transformed to a single-hidden-layer neural network training problem. There will be a preliminary study on the carefully selected method for each stage, and principles of related methods will be also introduced.

### 3.1. Problem Statement

The *n*th sample of a power signal with sampling rate $f_{s}$ can be expressed by a multisine waveform:

(1) $$y(n)=\sum_{m=1}^{M}A_{m}\sin\left(2\pi nf_{m}/f_{s}\;+\varphi_{m}\right)+\varepsilon \left(n\right)$$

where $f_{m}$ is the frequency of the *m*th harmonic/interharmonic component, $A_{m}$ and $\varphi_{m}$ are its corresponding amplitude and phase angle, and ε(n) is the white Gaussian noise with zero mean. The goal of harmonic and interharmonic estimation is to calculate the frequency, amplitude and phase angle of every component in an accurate and fast way.

As the sampled power signal sequence y(n) = ${\left[y(n),y(n-1),\ldots ,y(n-N+1)\right]}^{T}$ with dimension *N* is formed by a linear combination of the fundamental frequency component and its harmonics/interharmonics, and harmonics/interharmonics meet the condition that they are orthogonal and thus statistically independent of each other, extracting harmonic/interharmonic components from a single measurement channel can be treated as an extreme case of an underdetermined ICA problem. It is also known as single-channel independent component analysis. The work [34,44] explains when and how standard ICA can perform source separation from a single sensor and how SCICA can be applied in the analysis of electroencephalogram (EEG) and electrocardiograph (ECG) signals. The work [29,30] applies SCICA for harmonic component extraction from power system signals. Referring to the above works, an improved SCICA is proposed in this paper to speed up the algorithm and is proven robust enough to Gaussian noise.

In order to achieve resolution-enhanced frequency analysis at the expense of little computation, DFT-based high-resolution methods are preferred. In this paper, once single-frequency waveforms are obtained, the resolution-enhanced frequency can be directly calculated from only three DFT samples, the related signal processing techniques are presented in detail in [36,37]. Notably, it can be proved that the algorithm needs little additional computation and is robust enough to noise aiming at single-frequency signals. With the high-resolution frequencies and the original signal, the final problem is how to determine the amplitude and phase angle of each frequency component accurately in real-time. ADALINE has been widely applied in the harmonic estimation due to its self-adaption to noise and rapid convergence. In addition, the traditional ADALINE’s shortcoming can be overcome by the integration of proper frequency estimation methods just like the work in [21]. Therefore, ADALINE is a good choice for amplitude and phase angle estimation.

### 3.2. Principle of Single-Channel Independent Component Analysis

Independent component analysis (ICA) is a widely-used blind source separation method and can estimate independent sources based on the model of the mixture without any further prior knowledge. The process of applying single-channel independent component analysis (SCICA) to single-sensor signals can be regarded as adding harmonic/interharmonic extraction filter to a time-delayed model of signals.

In order to construct a time-delayed model for the ICA problem, the observed time series y(n) with dimension *N* can be separated into a sequence of contiguous blocks:

(2) $$x\left(n\right)={\left[y(n),y(n-1),\ldots ,y(n-D+1)\right]}^{T}$$

where *D* is the number of the blocks, and the superscript *T* represents transposition. x as the mixed signal matrix can be decomposed into the matrix product of mixing matrix and independent sources:

(3) x(n)=Es(n)

where $E_{D\times D}$ denotes the mixing matrix that linearly combines the sources to form a mixture of harmonics and interharmonics, s is a D×N matrix comprising the statistically independent sources such as pure sine waveforms with disjoint frequency spectra. To estimate the s from x, the inverse equation can be expressed by

(4) $$\hat{s}\left(n\right)=H\cdot x\left(n\right)$$

where $H=E^{-1}$ is the separation matrix, and $\hat{s}$ is the estimation of the original sources.

Thus, a standard ICA algorithm can be applied to x(n) and learn the sparse features of the measured signal, and the most common method to obtain the separation matrix is the FastICA algorithm. FastICA uses nonGaussianity as the statistical property of the signals for source separation, and Kurtosis is one of the classic measures for nonGaussianity estimation of zero-mean random variables. For better estimation, x should be preprocessed using principal component analysis or Whitening technique to make all the vectors of x have unit variance and uncorrelated.

To optimize the ICA algorithm, the fixed-point and gradient algorithm based on Kurtosis have been proposed in the literature. The kurtosis is the fourth standardized moment and also one of the classic measures of whether there are problems with outliers in a data set, namely, the estimation of non-Gaussianity for ICA. However, the presence of sub-Gaussian or super-Gaussian sources brings increased estimation error and computational complexity to the algorithms, and saddle points and spurious local extrema in the contrast functions are also not considered. Therefore, referring to the RobustICA algorithm [35] in this work, the Kurtosis contrast function is used as the objective function, and the optimal step size technique is integrated to complement the cost efficiency and robustness. In addition, preprocessing techniques are unnecessary, which simplifies the process.

### 3.3. Principles of ADALINE

ADALINE is a single-hidden-layer neural network with a linear transfer function and has been extensively used in signal processing, control systems and error cancellation. Recently, this technique also emerges in the harmonic and interharmonic analysis [11,21,45]. Regardless of the zero-mean noise, the estimated signal $\hat{y}(n)$ with known fundamental frequency and unknown amplitudes, phase angles can be represented as:

(5) $$\hat{y}\left(n\right)=\sum_{m=1}^{M}\left[{\hat{A}}_{m}\cos{\hat{\varphi }}_{m}\sin\left(2\pi nmf_{0}/f_{s}\;\right)+{\hat{A}}_{m}\sin{\hat{\varphi }}_{m}\cos\left(2\pi nmf_{0}/f_{s}\;\right)\right]$$

Then, substitute ${\hat{A}}_{m}\cos{\hat{\varphi }}_{m}$, ${\hat{A}}_{m}\sin{\hat{\varphi }}_{m}$, $2\pi nmf_{0}/f_{s}$ with ${\hat{W}}_{2m-1}$, ${\hat{W}}_{2m}$, ${\hat{\theta }}_{m}$ so that

(6) $$\hat{y}\left(n\right)=\sum_{m=1}^{M}\left({\hat{W}}_{2m-1}\sin{\hat{\theta }}_{m}+{\hat{W}}_{2m}\cos{\hat{\theta }}_{m}\right)={\hat{W}}^{T}\cdot \hat{X}\left(n\right)$$

where ${\hat{A}}_{m}$ and ${\hat{\varphi }}_{m}$ denote the estimated amplitudes and phase angles of the signal, and $\hat{W}={\left[{\hat{W}}_{1}\;{\hat{W}}_{2}\;\cdot \cdot \cdot \;{\hat{W}}_{2m-1}\;{\hat{W}}_{2m}\right]}^{T}$, $\hat{X}\left(n\right)={\left[\sin{\hat{\theta }}_{1}\;\cos{\hat{\theta }}_{1}\;\cdot \cdot \cdot \;\sin{\hat{\theta }}_{m}\;\cos{\hat{\theta }}_{m}\right]}^{T}$.

Based on each given input and target vector, the adaptive linear neuron network adjusts the weights and biases at each time step to minimize the sum-squared error of recent input and target vectors. The amplitude and phase of the *m*th frequency component are expressed by:

(7) $${\hat{A}}_{m}=\sqrt{{\left({\hat{W}}_{2m}\right)}^{2}+{\left({\hat{W}}_{2m-1}\right)}^{2}}$$

(8) $${\hat{\varphi }}_{m}=\tan^{-1}\left({\hat{W}}_{2m}/{\hat{W}}_{2m-1}\;\right)\;\;\;-\pi \leq {\hat{\varphi }}_{m}\leq \pi$$

Traditional ADALINE is only capable of computing the amplitudes and phase angles with known frequency as above. However, the presence of fundamental frequency deviation and interharmonics is usually not negligible in the practical situation, and it will cause serious interference to the convergence speed and accuracy of traditional ADALINE. Therefore, in this paper, the frequency deviation and interharmonics have been considered as the power signal is modeled, and gradient- or Jacobian-based methods have been tested for training the adaptive linear neuron network.

## 4. Proposed Method for Harmonics and Interharmonics Measurement

In this section, detailed description and improvement of the proposed method will be presented based on the preliminaries. It will also be discussed how the proposed method obtains a better fusion of harmonic and interharmonic extraction, frequency estimation, amplitude and phase angle estimation. In the end of this section, the overall process for the measurement of harmonic and interharmonics will be sketched.

### 4.1. Harmonic and Interharmonic Extraction

In this paper, unlike methods that use the FastICA algorithm to obtain the separation matrix, the algorithm named RobustICA is tested to achieve better performance on convergence speed, and sphering and whitening is not required as preprocessing as opposed to most ICA algorithms. The RobustICA technique is based on optimization of the Kurtosis contrast function, and it uses the optimal step-size technique to optimize the Kurtosis in the search direction. In particular, kurtosis contrast can be used in the source extraction with super-Gaussian sources included. Kurtosis is one of the classic measures for estimation of non-Gaussianity for ICA. Denote the Kurtosis of *α* by Kurt(α) and it is defined by:

(9) $$Kurt\left(\alpha \right)=\frac{E\left[\alpha^{4}\right]-2E^{2}\left[\alpha^{2}\right]-|E\left[\alpha^{2}\right]{|}^{2}}{E^{2}\left[\alpha^{2}\right]}$$

where E[·] denotes the mathematical expectation. Due to the coefficients such as step size, the learning rate in the iteration process directly affects the convergence speed, and the balance between convergence speed and accuracy is highly dependent on the step size. The optimal step size that maximizes the absolute Kurtosis function based on exact line search can be represented by

(10) $$\mu_{opt}=\arg\max_{\mu }\;\left|Kurt\left(h+\mu g\right)\right|$$

where h is a separating vector, and the search direction g is typically the gradient $g=\nabla_{w}Kurt\left(h\right)$. One iteration of RobustICA performs an optimal step-size optimization as follows:

*step* 1: The optimal step-size polynomial coefficient is given by $p\left(\mu \right)=\sum_{i=0}^{4}a_{i}\mu^{i}$, ${\left\{a_{k}\right\}}_{k=0}^{4}$ can be obtained from the measured signal and the values of g and h.
*step* 2: Extract optimal step-size polynomial roots ${\left\{\mu_{i}\right\}}_{i=0}^{4}$.
*step* 3: Select the root $\mu_{opt}$ leading to the absolute maximum of the contrast along the search direction.
*step* 4: Update $h=h+\mu_{opt}g$.

The stopping criterion for extracting one component is similar to that of FastICA. However, due to not applying the dimension reduction tricks like PCA, after every harmonic/interharmonic related component is extracted, the iterations for the remaining components will be a waste of time. In order to speed up this process, and also for the convenience of frequency estimation in the next stage, an additional stopping criterion is set according to the spectral energy ratio of each extracted component.

The proposed method SC-RICA is summarized as follows:

*step* 1: Construct the SCICA model.
*step* 2: Update the separating vector h iteratively using the optimal step-size method as mentioned.
*step* 3: Extract harmonic or interharmonic component $\hat{s}=h\cdot x$ with the optimized separating vector.
*step* 4: Calculate the DFT coefficients S of the extracted $\hat{s}$ and estimate the spectral energy ratio around the grid $k_{p}$ with the peak value of the magnitude.
*step* 5: If the spectral energy ratio exceeds the empirical threshold, loop from *step* 2. If not, break the loop and output $\hat{s}$, S, $k_{p}$ of every extracted component.

### 4.2. Frequency Estimation

The *m*th extracted harmonic/interharmonic component is close to the single-tone waveform observed under white Gaussian noise

(11) $${\hat{s}}_{\;}^{\left(m\right)}\left(n\right)=A^{\left(m\right)}\cos\left(2\pi nf_{m}/f_{s}\;+\varphi^{\left(m\right)}\right)+\varepsilon_{ext}\left(n\right)$$

where the superscript (m) indicates the index of the extracted component. The parameter of a sinusoidal waveform observed under white Gaussian noise is typically estimated with a coarse to fine strategy. First, DFT coefficients without interpolation are calculated for a coarse frequency estimate and the peak value of them is determined. Then, a resolution-enhanced search around the peak is provided.

Assuming the frequency of the extracted component is

(12) $$f_{m}=f_{s}\left(k_{p}+\delta \right)/N$$

where $k_{p}$ is the index of the maximum DFT magnitude coefficient and |δ|<1/2 represents the space between the true frequency point and the nearest discrete grid point. *N* is the sample size of the signal. The target at this stage is to estimate *δ* from samples around the peak in the DFT spectrum.

As mentioned above, after calculating the DFT coefficients of a single-tone waveform observed under white Gaussian noise, most of the signal energy will focus around the grid of $k_{p}$ in the Fourier domain due to spectral leakage. Additionally, $f_{m}$ generally is not exactly at the grid of $k_{p}$ unless $f_{m}$ is a multiple of the frequency resolution $f_{s}/N$.

However, leveraging the information around the grid of $k_{p}$ such as its neighbor $k_{p-1}$ and $k_{p+1}$, a more accurate frequency estimation will be obtained. The method proposed by Candan [37] is summarized as below:

*step* 1: Calculate the windowed DFT coefficients of the extracted harmonic/interharmonic component:
  (13) $$S_{k}=\sum_{i=0}^{N-1}w_{i}{\hat{s}}^{\left(m\right)}e^{-\frac{j2\pi ik}{N}},k=\left\{0,1,2,\ldots ,N\right\}$$
*step* 2: Find the index of the maximum DFT magnitude coefficient:
  (14) $$k_{p}=\underset{k=1,2\ldots N/2\;}{\arg\max}\;\left|S_{k}\right|$$
*step* 3: Select a window and calculate the window dependent function:
  (15) $$\begin{array}{c} f_{w}\left(\alpha \right)=\sum_{n=0}^{N-1}w\left[n\right]e^{j(2\pi nN)\alpha } \end{array}$$
  (16) $$\begin{array}{c} {f^{\prime }}_{w}\left(\alpha \right)=\frac{j2\pi }{N}\sum_{n=0}^{N-1}nw\left[n\right]e^{j(2\pi n/N)\alpha } \end{array}$$
  where w(n) is the real-valued window, and ${f^{\prime }}_{w}\left(\alpha \right)$ is the first derivative of $f_{w}\left(\alpha \right)$.
*step* 4: Calculate the bias correction factor of the selected window function:
  (17) $$c_{N}=\frac{Q_{0}^{2}}{P_{1}Q_{0}+P_{0}Q_{1}}$$
  where
  (18) P0=Imag{fw(1)−fw(−1)}
  (19) $$\begin{array}{c} P_{1}={f^{\prime }}_{w}\left(1\right)-{f^{\prime }}_{w}\left(-1\right) \end{array}$$
  (20) $$\begin{array}{c} Q_{0}=2f_{w}\left(0\right)-f_{w}\left(1\right)-f_{w}\left(-1\right) \end{array}$$
  (21) Q1=Imag{2f′w(0)}−f′w(1)−f′w(−1)}
*step* 5: Estimate *δ* using the following equation:
  (22) $$\hat{\delta }=c_{N}\text{Real}\left\{\frac{S_{k_{p}-1}-S_{k_{p}+1}}{2S_{k_{p}}-S_{k_{p}-1}-S_{k_{p}+1}}\right\}$$
*step* 6: Estimate $f_{m}$ of the extracted component with the function given in Equation (12).

The result of the first two steps has been already obtained in the stage of harmonic and interharmonic extraction. Further proof and derivation can be found in [36,37]. The method of this stage obtains resolution-enhanced frequency estimation without much additional computation, and it been proved robust in the presence of noise. Due to some interfering components that may be not filtered out completely, the application of the window function alleviates the problem to some extent.

### 4.3. Amplitude and Phase Angle Estimation

Once all the high-resolution frequency components are obtained, the estimation of amplitudes and phase angles can be considered as learning a linear or nonlinear mapping between appropriate inputs and outputs. Unlike traditional ADALINE, the fundamental frequency deviation and interharmonics have been considered at this stage. Regardless of the zero-mean noise, the estimated signal $\hat{y}$ can be represented as:

(23) $$\hat{y}\left(n\right)=\sum_{m=1}^{M}\left[{\hat{A}}_{m}\cos{\hat{\varphi }}_{m}\sin\left(2\pi n{\hat{f}}_{m}/f_{s}\;\right)+{\hat{A}}_{m}\sin{\hat{\varphi }}_{m}\cos\left(2\pi n{\hat{f}}_{m}/f_{s}\;\right)\right]$$

Then, substitute ${\hat{A}}_{m}\cos{\hat{\varphi }}_{m}$, ${\hat{A}}_{m}\sin{\hat{\varphi }}_{m}$, $2\pi n{\hat{f}}_{m}/f_{s}$ with ${\hat{W}}_{2m-1}$, ${\hat{W}}_{2m}$, ${\hat{\theta }}_{m}$, so that

(24) $$\hat{y}\left(n\right)=\sum_{m=1}^{M}\left({\hat{W}}_{2m-1}\sin{\hat{\theta }}_{m}+{\hat{W}}_{2m}\cos{\hat{\theta }}_{m}\right)={\hat{W}}^{T}\cdot \hat{X}\left(n\right)$$

where ${\hat{f}}_{m}$, ${\hat{A}}_{m}$, ${\hat{\varphi }}_{m}$ denote the estimated frequencies, amplitudes and phase angles of the signal, and $\hat{W}={\left[{\hat{W}}_{1}\;{\hat{W}}_{2}\;\cdot \cdot \cdot \;{\hat{W}}_{2m-1}\;{\hat{W}}_{2m}\right]}^{T}$, $\hat{X}\left(n\right)={\left[\sin{\hat{\theta }}_{1}\;\cos{\hat{\theta }}_{1}\;\cdot \cdot \cdot \;\sin{\hat{\theta }}_{m}\;\cos{\hat{\theta }}_{m}\right]}^{T}$.

In this situation, the neural network architecture is constructed with $\hat{X}\left(n\right)$ as the input and the original signal *y* in the time domain as the target output. In addition, there is no bias connected to the network layers, and the transfer function is linear rather than hard-limiting, which allows its outputs to take on any value. The mean squared normalized error with regularization can be taken as the cost function

(25) $$J\left(\hat{W}\right)=\left[\frac{1}{N}\sum_{i=1}^{N}\left(\frac{1}{2}{\left\|\hat{W}\hat{X}\left(i\right)-y_{i}\right\|}^{2}\right)\right]+\frac{\lambda }{2}\sum_{i=1}^{2M}{\left({\hat{W}}_{i}\right)}^{2}$$

where the subscript *i* indicates the index of a vector element, *N* denotes the sample size of the power signal, *M* denotes the number of frequency components, and *λ* is the weight decay parameter that controls the relative importance of the two terms in the definition of $J(\hat{W})$.

Our goal is to minimize $J(\hat{W})$ as a function of $\hat{W}$. To train the neural network, we initialize each ${\hat{W}}_{i}$ to a small random value near zero, then update the weight values using some network training function, such as gradient- or Jacobian-based methods. As the network’s weight, input and transfer functions have derivative functions, a scaled conjugate gradient backpropagation is used to calculate derivatives of the cost function with respect to the weight variable.

One iteration of gradient descent updates the $\hat{W}$ as follows:

(26) $${\hat{W}}_{i}:={\hat{W}}_{i}-\beta \frac{\partial }{\partial {\hat{W}}_{i}}J\left(\hat{W}\right)$$

(27) $$\alpha \frac{\partial }{\partial {\hat{W}}_{i}}J\left(\hat{W}\right):=\left[\frac{1}{N}\sum_{i=1}^{N}\beta \frac{\partial }{\partial {\hat{W}}_{i}}J\left(\hat{W};X\left(i\right),y\left(i\right)\right)\right]+\lambda {\hat{W}}_{i}$$

where *β* denotes the learning rate.

By repeatedly taking steps of gradient descent to reduce the cost function $J(\hat{W})$, and setting appropriate stopping criterion, the neural network will be well trained and W will contain the information of amplitudes and phases. Then, the amplitude and phase of the *m*th frequency component are expressed by

(28) $${\hat{A}}_{m}=\sqrt{{\left({\hat{W}}_{2m}\right)}^{2}+{\left({\hat{W}}_{2m-1}\right)}^{2}}$$

(29) $${\hat{\varphi }}_{m}=\tan^{-1}\left({\hat{W}}_{2m}/{\hat{W}}_{2m-1}\;\right)\;\;\;-\pi \leq {\hat{\varphi }}_{m}\leq \pi$$

This hybrid method consists of three stages: harmonic and interharmonic extraction, frequency estimation, amplitude and phase angle estimation. Extracting pure sinusoids is the guarantee of the resolution-enhanced frequency estimation, and frequency estimation is an important factor to accurate harmonic and interharmonic measurement. In summary, the overall process for the measurement of harmonics and interharmonics can be sketched as follows:

*step* 1: Construct the time-delayed model as the input of SC-RICA.
*step* 2: Extract the harmonic/interharmonic components using SC-RICA.
*step* 3: Output the DFT coefficients of each extracted component and the index with the maximum magnitude to the next step.
*step* 4: Estimate the high-resolution frequency of each component from three DFT samples.
*step* 5: Take the estimated frequency as the input of ADALINE, and the original signal as the output. Train the neural network until meeting the stopping criterion.
*step* 6: Calculate amplitudes and phase angles of extracted components of the measured power signal.

## 5. Performance Evaluation

The experiments are organized as follows: Section 5.1: synthesized power signals including harmonics, interharmonics are analyzed, fundamental frequency deviation and noise are also considered. Section 5.2: PWM (pulse width modulation) VSI (voltage source inverter) induction motor drives as loads in an IEEE 14 bus system are simulated by SimPowerSystems^TM^ (MathWorks, Natick, MA, USA), and the emitted harmonic and interharmonic disturbances are measured and then analyzed. Section 5.3: laboratory experiments are conducted on a prototype system which is designed according to the proposed power quality analysis framework in networked environment. At the moment of switching, the microgrid from grid-connected mode to isolated mode, and the estimation results of the fundamental frequency variation are analyzed and compared in the presence of harmonics. In addition, field experimental signals with harmonic and interharmonic currents injected by regularly fluctuating loads such as laser printers are acquired and analyzed. Section 5.4: the results of simulation and field experiments are analyzed, and the characteristics of the proposed method are also discussed.

### 5.1. Measurement of Synthesized Harmonics and Interharmonics with Frequency Deviation and Noise

In order to illustrate the performance of the proposed method, synthesized power signals including harmonics and interharmonics are generated, and the methods are implemented in MATLAB with a desktop personal computer having an Intel Core i5-3470 processor ( Intel Corporation, Santa Clara, CA, USA) and an 8-GB random access memory. The parameters of the signal are listed in Table 1. There are eight components represented as component numbers from one to eight, and they are spectrally disjoint. It can be seen from Table 1 that the fundamental frequency deviation has been considered within a 50 Hz power system, and 0.1 Hz as the fundamental frequency deviation is an acceptable assumption. The synthesized power signals mainly consist of fundamental, 3rd, 5th harmonics and five interharmonic components around them. To ensure that the synthesized signals are close to real power signals, the amplitudes of harmonics and interharmonics range from around 3% to 60% of the fundamentals.

For comparison, white Gaussian noises with zero mean are randomly generated and added to the pure synthesized signals, and the signal to noise ratio (SNR) is set to 40 dB. *K* synthesized signals have been sampled with size *N* = 1000 and sampling frequency 5 kHz. Three methods are chosen: EMO-ESPRIT [33], MUSIC [25], and CS-DFT [23]. As the computation times of all four methods have positive correlations with the number of harmonic and interharmonic components in the synthesized signals, and all the synthesized signals are composed of eight harmonic/ interharmonic components, for a fair and reasonable comparison, the model orders of MUSIC and EMO-ESPRIT are strictly set to 16, and the dimensions of the autocorrelation matrix are tuned to be as small as possible without sacrificing the estimation accuracy. Similarly, CS-DFT terminates the algorithm when the iteration number of the support recovery exceeds 16, and the interpolation factor of CS-DFT is set to 10 as [23] suggested. In terms of the proposed method, it stops the harmonic extraction when 16 components have been extracted and terminates the adaptive linear neural network training when the gradient is below 1.00 × 10^−6^.

**Figure 2:**
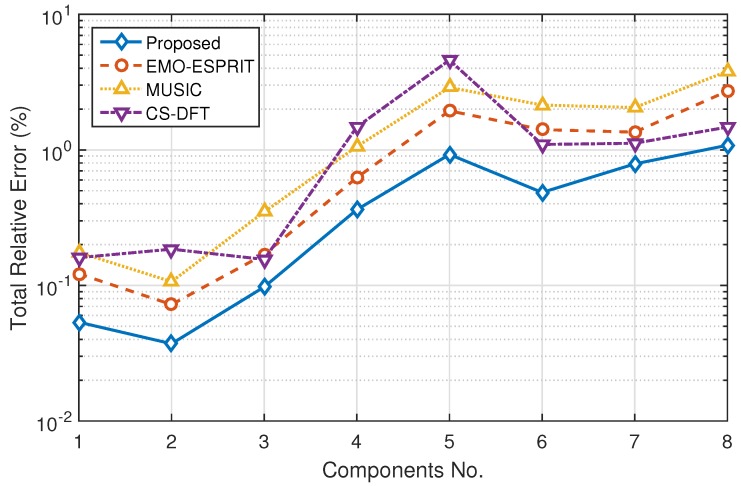
Total relative errors of amplitude estimation.

The tested signals in Section 5.1 are synthesized with the parameters in Table 1 so that the actual values of parameters such as amplitudes and phase angles are known. Therefore, we can use the difference between the actual and estimated value to evaluate the algorithms. The total relative errors (TRE) of amplitude estimation is used for performance evaluation and defined as

(30) $$\text{TRE}=\sum_{k=1}^{K}\left(\frac{|A_{\text{actual}}\left(k\right)-A_{\text{estimated}}\left(k\right)|}{A_{\text{actual}}\left(k\right)}\times 100\%\right)/K$$

where $A_{\text{actual}}\left(k\right)$ means the measured component’s amplitude of the *k*th sampled signal. The total relative errors of amplitude estimation using different methods are depicted in Figure 2. It can be observed that the proposed method achieves better amplitude estimation than the others, and its TRE varies from 0.04% to 1.08% in this case, and the value of TRE is highly related to the amplitude ratio of harmonic to fundamental, the TRE of harmonics and interharmonics with relatively high ratio is small, and vice versa. However, in the presence of interharmonics and frequency deviation, the performance of CS-DFT is less stable than others, as the actual frequency component is not lying on the fine grids in the frequency domain. The average computation times of EMO-ESPRIT, MUSIC, CS-DFT and the proposed method are also shown in Table 2.

To evaluate the amplitude estimation accuracy under different signal-to-noise ratios (SNRs), the algorithms have also been implemented on the system constructed as follows. The signals are synthesized using the parameters given in Table 1 on the personal computer. Then, the arbitrary function generator AFG3102 (Tektronix, Beaverton, OR, United States) receives the instructions through ethernet and outputs the signals with additive zero mean Gaussian white noise. Noisy signals are collected by oscilloscope DSO7032B (Agilent, Santa Clara, CA, United States) via BNC (Bayonet Neill-Concelman) connector and sent to another PC for subsequent processing.

**Figure 3:**
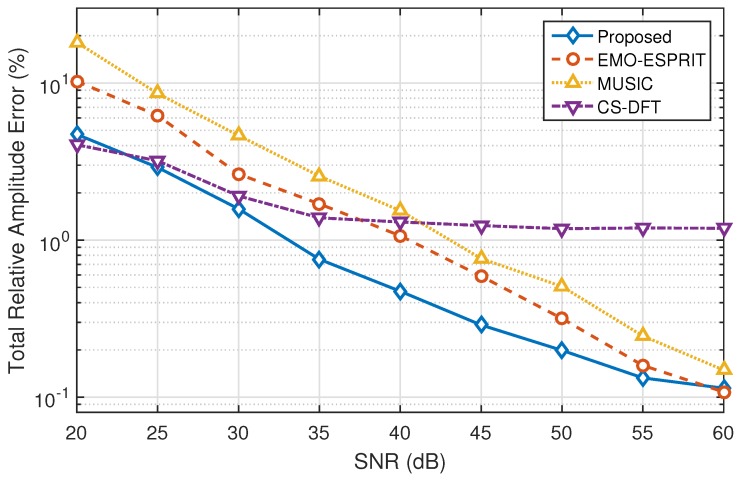
The total relative error of amplitude estimation under different signal-to-noise ratios.

The signals of each signal-to-noise ratio have been acquired 100 times, and the total relative amplitude error in this case is defined as the total relative error of the average amplitude of all the frequency components. The total relative amplitude errors calculated using EMO-ESPRIT, MUSIC, CS-DFT and the proposed method under different signal-to-noise ratios are depicted in Figure 3. According to IEC standard 61000-4-7 [8], the signal-to-noise ratio is limited to not below 20 dB in the power system, so that the SNR in this case is set from 20 dB to 60 dB. It can be noticed that the total relative amplitude errors of EMO-ESPRIT, MUSIC and the proposed method significantly decrease as the SNR increases, although the proposed method does not obviously overmatch the other two methods when the SNR is beyond 60 dB and the noise is negligible. The proposed method is much more robust when the SNR is between 20 dB and 60 dB, and SNR in this range is very common in ordinary power systems. On the contrary, due to the relatively low frequency resolution of CS-DFT, which is restricted by the discretized and uniformly-spaced frequency grid, the presence of fundamental frequency deviation and interharmonics causes inevitable amplitude estimation error to CS-DFT results. Although CS-DFT has the potential to deal with low-SNR power signals, the frequency obtained by support recovery will not fall on the nearest frequency grid with high probability if the SNR is below 20 dB, and thus the performance of frequency estimation will be significantly weakened.

### 5.2. Simulation Results from the PWM VSI Induction Motor Drive

Adjustable speed drives are widely used in various industries and can be regarded as main loads, adjustable speed drives usually utilize interlinked frequency converters which are just the sources of interharmonics. These interharmonics vary with the load frequency due to the propagation of load current into the DC (direct current) link. To evaluate the proposed method on experimental signals closer to operating conditions, a PWM (pulse width modulation) VSI (voltage source inverter) induction 3HP motor drive as a load in an IEEE 14 bus system is built using SimPowerSystem^TM^, and the interharmonics produced by PWM adjustable speed drive are considered not negligible. The induction motor drive mainly consists of a three-phase AC (alternating current) supply, a PWM inverter which is built using a universal bridge, an induction motor driving a mechanical load and a three-phase diode rectifier converting AC to DC. The simulation of this system is discretized with a 2 *μ*s time step and lasts for 1 second, and the motor speed set point is 1000 rpm. Specific simulation parameters are shown in Table 3.

**Figure 4:**
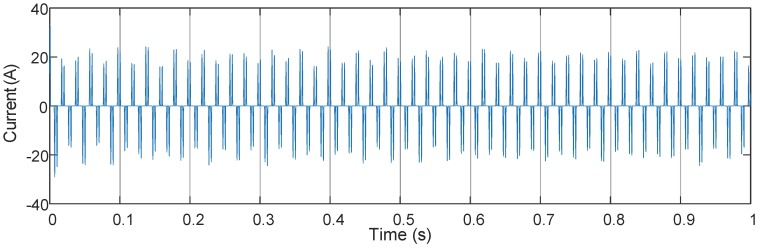
Measured current waveform of the induction motor drive. 0–1s: steady operation condition.

**Figure 5:**
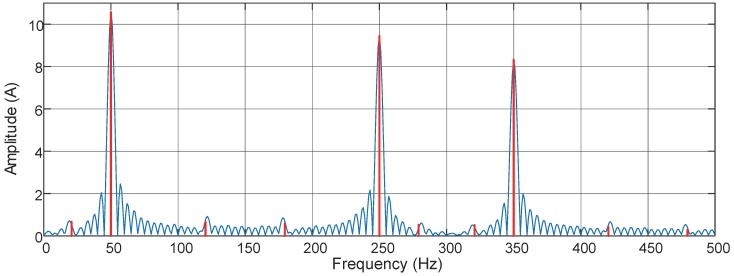
Calculated current spectrum of the induction motor drive with the proposed method. A zero-padded discrete Fourier transform (**blue**) is plotted for comparison.

The distorted signal depicted in Figure 4 is captured at the steady operation period, after reducing the sampling points through downsampling, and the data sampled at 5 kHz is sent to the algorithms. The resolution-enhanced harmonic and interharmonic measurement techniques such as MUSIC, EMO-ESPRIT, CS-DFT and the proposed method generally assume that the distorted signals are sparse in frequency domain. According to the theoretical interharmonic frequency of the source current of this motor drive [13], the current signals captured at the steady operation period can be considered to be sparse with line spectrum. Because the frequency spectrum of the waveform at the acceleration period is not a line spectrum and of no concern in this paper, only the waveform at the steady operation period is set as input to algorithms. In order to be seen clearly, Figure 5 plots the frequency spectrum below 500 Hz and there are much more harmonic and interharmonic components not shown in the figure, and for simple comparison, a zero-padded DFT is plotted in the frequency spectrum.

As actual harmonic and interharmonic components in the signals of Section 5.2 are unknown, simply calculating the parameters such as amplitudes and phase angles for comparison is meaningless. Therefore, different from TRE, reconstruction error [9,22] of the measured signals as a more suitable candidate is used as the evaluation criterion

(31) $$\varepsilon_{r}=\sqrt{\frac{1}{N\cdot y_{rms}^{2}}\sum_{n=1}^{N}{\left[y_{re}\left(n\right)-y_{ms}\left(n\right)\right]}^{2}}$$

where $y_{re}$ is the signal reconstructed using the result produced by some method, $y_{ms}$ is the measured signal and $y_{rms}$ is the root mean square of $y_{ms}$.

The statistical characteristics of the reconstruction error computed from 100 random samples of the measured signals are shown in Table 4, and high-resolution methods such as CS-DFT, EMO-ESPRIT, MUSIC are used for comparison. Because the model order of the measured signal in this case is large and unknown, it is assumed that the model order for MUSIC is 100. As CS-DFT and the proposed method are based on iteration, only appropriate stopping criterion is set. The result shows that the proposed method outperforms the other methods in terms of the reconstruction error, and CS-DFT also performs well because the actual frequency components are just lying near the interpolated frequency grid, although with relatively low frequency resolution. However, EMO-ESPRIT and MUSIC achieve unsatisfactory performance because the order of the covariance matrix is hard to tune, and, for MUSIC, the estimated model order is different from the actual signals.

To fulfill the demands such as power quality analysis in appliance level and the interaction between computerized instrumentation and physical facilities, a deeper insight of the (inter) harmonic sources can also be obtained. The theoretical interharmonic frequency $f_{ih}$ of the source current of this motor drive is given by the following expression $f_{ih}=\left|\left(p_{1}m\pm 1\right)f_{1}\pm p_{2}nf_{2}\right|$, where m=0,1,2…, n=1,2,3…, $p_{1}=6$, $p_{2}=2$ are known a priori and denote the numbers of pulses of the rectifier and inverter, respectively, $f_{1}$ denotes the fundamental frequency of the supply, and $f_{2}$ is the load frequency. Therefore, the load frequency can be estimated from the frequency spectrum. From the spectrum presented in Figure 5, fundamental frequency, 5th, and 7th harmonics are the main components of this distorted signal, and there are interharmonics with frequencies of 120.5 Hz, 179.5 Hz, 279.5 Hz, 320.5 Hz, 420.5 Hz. $f_{1}=50.0\;\text{Hz}$ is the fundamental frequency; therefore, $f_{2}$, estimated with the above equation, is about 35.3 Hz and very close to the load frequency calculated from the stator current waveform. It can be seen that the accurate measurement of harmonic and interharmonic with the proposed method has revealed some intrinsic information of the electric equipment, and it provides useful knowledge for subsequent analyzing such as harmonic and interharmonic source location or fault location, and assists the control center to achieve a more sophisticated control for (inter)harmonic mitigation or even power quality enhancement. Conceivably, this kind of processing and analyzing techniques will be significantly beneficial for digital modernization.

### 5.3. Laboratory Experiments on the Prototype System

The prototype system for power quality analysis in networked environment has been built under the laboratory environment. The nonlinear loads are distributed according to the application scenarios. For instance, fluorescent lighting and uninterrupted power supply (UPS) systems form the main loads for commercial users, laser printers and personal computers are chosen as the loads in offices, and the loads of AC/DC rotors emerge for industrial users. Voltage and current signals of all the consumers are simultaneously acquired by a National Instruments (Austin, TX, USA) Ethernet RIO Expansion Chassis NI 9148 equipped with voltage modules (National Instruments NI 9225, 50 kS/s sample rate, 300 Vrms measurement range, 24-bit resolution) and current modules (National Instruments NI 9227, 50 kS/s sample rate, 5 Arms measurement range). The signals are further processed by intelligent information processing techniques, and then the harmonic and interharmonic estimation results are transmitted through ZigBee network, and CC2430 from Texas Instruments (Dallas, TX, USA) is chosen as the core chip of the ZigBee end device. Given the possible power failure, the ZigBee end device can be powered by either an external power supply or an internal battery. All of the power quality data in the laboratory will be aggregated to ZigBee coordinators and further processed in the control center deployed on a personal computer. Furthermore, the control center can also be considered to be an information publishing platform that is capable of announcing power-quality-related alerts, electricity usage, price and so on. In this prototype, web services for obtaining the harmonic and interharmonic estimation results are deployed on the Internet, and for demonstration purposes, visualized results are depicted on a remote tablet computer (Nexus 10, Google, Mountain View, CA, USA) in real-time. All of above components constitute the prototype for power quality analysis in networked environment, and the laboratory experiments as follows are conducted in this prototype system.

The first result discussed in this section refers to the estimation of the fundamental frequency variation in the presence of harmonics. This experiment is conducted in the prototype system mentioned above, and a SANTAK (Shenzhen, China) C3K UPS serves as a standby. Initially, the microgrid where a DC motor is running is connected to the power grid. Then, the external supply is interrupted around the time of 4.0 s. Finally, the UPS serves as the main supply to the end.

**Figure 6:**
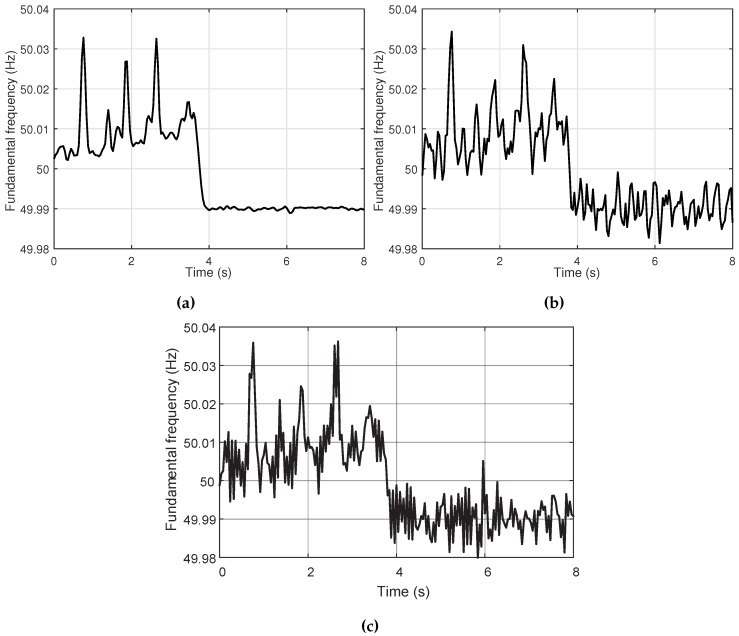
Estimation of the fundamental frequency variation in the presence of harmonics using (**a**) the proposed method; (**b**) EMO-ESPRIT; and (**c**) MUSIC.

To capture the slight variation of the fundamental frequency under the distortion of harmonics, only current signal acquisition devices are deployed. To evaluate the estimation result in the presence of harmonics, the fundamental frequency of the distorted current signal is computed with the proposed method, EMO-ESPRIT and MUSIC. The result of CS-DFT is not depicted because the frequencies obtained by CS-DFT are discrete in a fine frequency grid, and the interpolation factor of CS-DFT is advised to be not much larger than the order of 10 in [23]; otherwise, the numerical conditioning tends to get worse. In this paper, the interpolation factor of CS-DFT is set to 10, which means its frequency resolution is 0.5 Hz. This resolution is inadequate for tracking the slight variation of fundamental frequency. Therefore, aiming at the estimation of the fundamental frequency variation, EMO-ESPRIT and MUSIC are chosen for comparison. The result is depicted in Figure 6. From the computed value of the fundamental frequency, it can be seen that the variations estimated by all methods are much more stable when the UPS takes over because the system of the microgrid is less complicated, and there is a sudden change at around 4.0 s just as the external supply interrupts. Because all the methods belong to batch processing techniques, the analyzed window length and moving step are set to 200 and 40 ms, respectively. Although the computed frequency varies slightly from about 49.99 Hz to 50.03 Hz, both EMO-ESPRIT, MUSIC and the proposed method track the actual frequency well with little deviation, and it can be inferred that the result of the proposed method is more in accordance with the ground truth.

The second result of this section refers to the parameter estimation of another important type of load. Experimental signals comprising harmonics and interharmonics are acquired and the laboratory setup is given in the prototype mentioned above. Apart from the PWM VSI induction motor drive, regularly fluctuating loads such as welder machines and laser printers can also be harmonic and interharmonic sources. Here, laser printers are used as the fluctuating loads in the system, the load is fed by a 220 V, 50 Hz single-phase AC supply with a miniature circuit breaker. For current waveform measurement, NI 9227 as a current input module is in series with the AC supply and the loads, and gives a multi-channel measurement of the waveforms. Then, an Ethernet RIO with an NI 9227 inserted sends the multi-channel waveform data to the power quality analysis units for subsequent processing.

**Figure 7:**
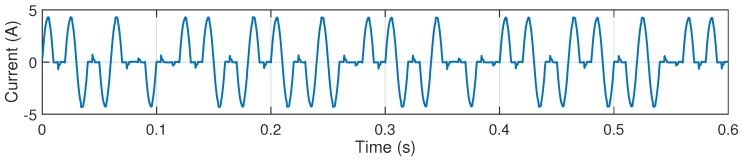
Measured current waveform of the regularly fluctuating load from the chosen channel.

**Figure 8:**
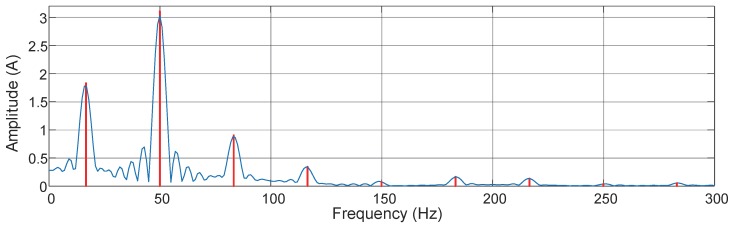
Calculated current spectrum of the regularly fluctuating load with the proposed method. A zero-padded discrete Fourier transform (**blue**) is plotted for comparison.

The current waveform depicted in Figure 7 is a measured time waveform of the regularly fluctuating load from the chosen channel. It can be observed from the spectrum in Figure 8 that, firstly, the amplitudes calculated by the proposed method are not equal to the peak values of the zero-padded DFT. They are usually larger than the peak values due to the spectrum leakage. Secondly, the frequencies calculated by the proposed method are also not exactly on the discrete frequency grid of the zero-padded DFT. In addition, there are mainly fundamental, 3rd, and 5th harmonic components each with two interharmonics distributed around them, and the frequency distribution is similar to the mathematical model of the regular fluctuating load [13]. Thus, the modulation frequency can be inferred to be around 34.0 Hz. Apart from the applications presented in Section 5.2, non-intrusive appliance load monitoring is highly dependent on the information extracted from this waveform-level data which is also addressed for power quality analysis in networked environment, and the monitoring and control will be zoomed to the appliance level. It can be inferred that the success of this research will create truly smart PQAs or smart meters.

### 5.4. Discussion

Simulation and laboratory experiments have been conducted in this work. Apart from the enhanced resolution and accuracy shown in the experiments, some other technical characteristics should be noticed.

Compared to classic and state-of-art methods with high frequency resolution, the proposed method is remarkably accurate in parameter estimation. This proves that extracting harmonic and interharmonic components is beneficial to subsequent processing, and resolution-enhanced frequency can be acquired with only three DFT coefficients. Furthermore, the proposed method is relatively adaptable to noise and fundamental frequency deviation. It achieves the best performance under SNR between 20 dB and 60 dB among the four methods observed from Figure 3, and the noise in this range is quite common in electric power systems [8]. Although MUSIC, EMO-ESPRIT achieve better performance than the proposed method when SNR exceeds 60 dB and CS-DFT prevails when SNR is below 20 dB, they are not engineering-practical enough. This is mainly due to the careful selection and improvement of the method in each stage. In the harmonic and interharmonic extraction stage, unlike traditional ICA, sub-Gaussian or super-Gaussian sources are considered in SC-RICA. In the parameter estimation stage, DFT and ADALINE are robust to noise in nature. Furthermore, the frequency of the proposed method lies in the continuous domain. This provides a more precise result than CS-DFT in the presence of fundamental frequency deviation and interharmonics.

From the view of computational complexity, the proposed method is less demanding than other existing high-resolution methods, and utilizes the least computing resource among these four methods without sacrificing the accuracy. Relatively, MUSIC, EMO-ESPRIT and CS-DFT are still suitable for offline analysis, limited by their high computational burden [14,23]. As for the proposed method, although it is a hybrid, cascading method, it has been examined that one separation row can be achieved in only one iteration with the help of optimal step-size optimization in the harmonic and interharmonic extraction stage, and the separation results are fully acceptable for the subsequent processing. In addition, fine frequencies can be estimated with little additional computation using the method described in Section 4.2, and ADALINE can obtain a fast convergence in no more than 10 iterations. Specifically, the most computation-intensive part is the harmonic and interharmonic extraction. To speed up the convergence of ICA, the optimal step-size optimization is performed using an exact line search technique, although the computational complexity per iteration of RobustICA (5D·N+12N) is more than twice the FastICA (2D·N+2N). The convergence of RobustICA is remarkably faster than that of FastICA, and, generally, the acceptable separation vector can be achieved in no more than one iteration. In addition, ADALINE and FFT are highly parallelizable tasks, and they can both be implemented efficiently on a field-programmable gate array (FPGA). In contrast, the iterative greedy support recovery of CS-DFT adds too much additional computation, and the computation times of subspace methods such as MUSIC and EMO-ESPRIT highly depend on the dimension of the covariance matrix.

However, some restrictions have been found during the experiments. The individual components are extracted iteratively and the ADALINE network’s size and convergence speed are dependent on the number of components. Therefore, the proposed method still needs much time when there are excessive harmonic and interharmonic components. Furthermore, the proposed method is a batch processing technique and mainly focuses on the power signals with a line spectrum. More precisely, short-term steady-state measurements of the harmonic and interharmonic is a concern in this paper. From the experiment results, it could be inferred that the proposed method is appropriate to be implemented on power quality analyzers for high-resolution analysis of harmonics and interharmonics, and after fine-grained information being accurately extracted, many power-quality-related applications will profit in this networked environment for power quality analysis.

## 6. Conclusions

In this paper, power quality issues in a cyber-physical energy system have been addressed and a resolution-enhanced approach to harmonic and interharmonic estimation has been proposed for power quality analysis in networked environment. Considering that microgrids are widespread, and with higher harmonic and interharmonic distortion in the future, a power quality analysis framework is designed deploying power quality analyzers closer to the consumers for lower-level sensing and control, and the wireless sensor network is also emphasized for more effective data transmission. In such a framework, the proposed method utilizes the single-channel version of RobustICA to extract harmonic and interharmonic components in a time-efficient way. Then, high-resolution frequencies are obtained from three DFT samples with little additional computation. Finally, amplitudes and phases are calculated with the ADALINE network to improve the adaptivity to noise. The proposed method has been tested on synthetic and experimental harmonic and interharmonic signals, and accurate amplitude estimation and resolution-enhanced frequency estimation can be achieved time-efficiently in the presence of noise and fundamental frequency deviation. From the view of accuracy and computational complexity, the proposed method obtains a better tradeoff compared to the existing methods, and it is more suitable to be implemented in hardware for high-resolution measurement of harmonic and interharmonic. Although the proposed method reduces the computational burden considerably, the computation time is still much higher than most of the DFT-based techniques. Therefore, future work will be focused on the further reduction of the computational time.

## Figures and Tables

**Table 1:** Parameters of the synthesized signal with eight components.

Component No.	1	2	3	4	5	6	7	8
*f* (Hz)	16.4	49.9	83.7	116.1	149.7	183.2	216.3	249.5
*A* (pu)	1.80	3.10	0.90	0.30	0.10	0.16	0.13	0.10
*ϕ* (rad)	$-\frac{\pi }{2}$	$-\frac{\pi }{2}$	0	$\frac{\pi }{2}$	$\frac{\pi }{2}$	0	$\frac{3\pi }{4}$	$-\frac{\pi }{4}$

**Table 2:** Comparison of computation times.

	CS-DFT	EMO-ESPRIT	MUSIC	Proposed
Average Computation Time (ms)	487.2	116.3	529.8	64.1

**Table 3:** Simulation parameters.

AC Supply				PWM Inverter	
Source voltage	Power frequency	Source resistance	Source inductance	Device type	Carrier frequency
220 V	50 Hz	0.02 *Ω*	0.05 mH	IGBT/Diodes	4.5 kHz
**Induction Motor**					**DC Link**
Rated power	Rated voltage	Operating speed	Equivalent resistance	Equivalent Inductance	Capacitance
2238 VA	220 V	1000 rpm	0.435 Ω	2 mH	3400 *μ*F

**Table 4:** Reconstruction error of the measured signals for Section 5.2.

Reconstruction Error	CS-DFT	EMO-ESPRIT	MUSIC	Proposed method
Mean	3.8 × 10^−2^	4.9 × 10^−2^	4.6 × 10^−2^	2.4 × 10^−2^
Max	3.9 × 10^−2^	5.0 × 10^−2^	4.7 × 10^−2^	2.9 × 10^−2^
Standard Deviation	6.5 × 10^−2^	5.1 × 10^−2^	2.1 × 10^−2^	7.0 × 10^−2^
